# Efficient All-Polymer Solar Cells Enabled by Interface Engineering

**DOI:** 10.3390/polym14183835

**Published:** 2022-09-14

**Authors:** Guoping Zhang, Lihong Wang, Chaoyue Zhao, Yajie Wang, Ruiyu Hu, Jiaxu Che, Siying He, Wei Chen, Leifeng Cao, Zhenghui Luo, Mingxia Qiu, Shunpu Li, Guangye Zhang

**Affiliations:** 1 College of New Materials and New Energies, Shenzhen Technology University, Shenzhen 518118, China; 2 College of Engineering Physics, Shenzhen Technology University, Shenzhen 518118, China; 3 College of Materials Science and Engineering, Shenzhen University, Shenzhen 518060, China

**Keywords:** organic photovoltaics, all-polymer solar cells, power conversion efficiency, electron transport layer

## Abstract

All-polymer solar cells (all-PSCs) are organic solar cells in which both the electron donor and the acceptor are polymers and are considered more promising in large-scale production. Thanks to the polymerizing small molecule acceptor strategy, the power conversion efficiency of all-PSCs has ushered in a leap in recent years. However, due to the electrical properties of polymerized small-molecule acceptors (PSMAs), the FF of the devices is generally not high. The typical electron transport material widely used in these devices is PNDIT-F3N, and it is a common strategy to improve the device fill factor (FF) through interface engineering. This work improves the efficiency of all-polymer solar cells through interfacial layer engineering. Using PDINN as the electron transport layer, we boost the FF of the devices from 69.21% to 72.05% and the power conversion efficiency (PCE) from 15.47% to 16.41%. This is the highest efficiency for a PY-IT-based binary all-polymer solar cell. This improvement is demonstrated in different all-polymer material systems.

## 1. Introduction

**Figure 1:**
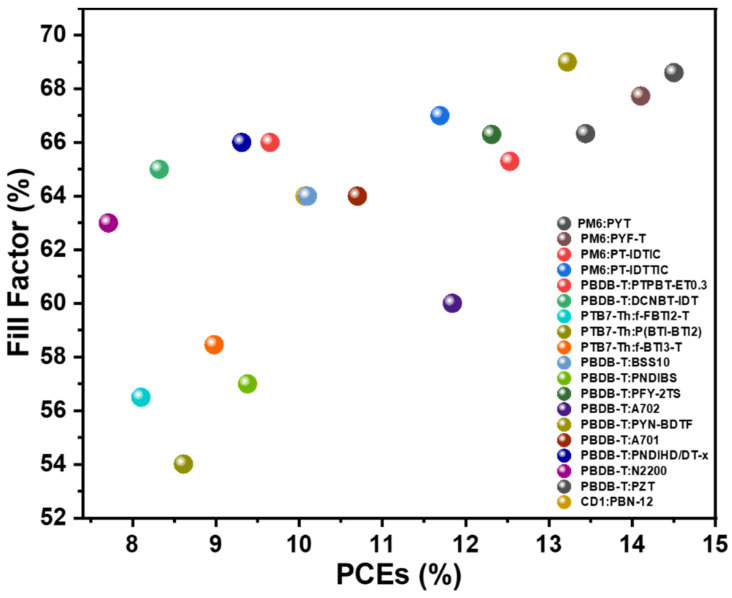
Fill Factors and PCEs for several high-performance all-polymer solar cells in recent years.

Organic solar cells (OSCs) are advantageous for distributed photovoltaic applications due to their flexibility, semitransparency, high indoor light matching, high power generation per unit weight, patternable design, etc. [1,2,3,4,5,6,7]. While the classical OSCs active layer consists of a polymer donor and a fullerene derivative acceptor [8], with the development of non-fullerene acceptor materials, the mainstream of research has now shifted to a material system based on a polymer donor and a non-fullerene acceptor [9,10,11,12,13,14,15,16,17]. Compared with fullerene acceptors, non-fullerene acceptors have flexible chemical structure designs and thus easily tunable optoelectronic properties, endowing them with more compatible absorbance spectra and electrical properties with the donor. Additionally, the resulting devices usually exhibit lower voltage losses [18], which improve device efficiency [19]. In terms of materials, non-fullerene acceptors are divided into two main categories: small-molecule acceptors and polymer acceptors. In contrast to the small molecule acceptor, when the acceptor is polymer, all-polymer solar cells (All-PSCs) can be prepared by combining with a polymer donor. In addition to similar advantages to small-molecule acceptor-based OSCs, All-PSCs possess better mechanical properties [20,21], such as higher tensile and flexural toughness, and potentially higher thermal stability, which provide them with better prospects for large-scale production [22]. The all-PSCs field has evolved over 20 years, starting with the earliest polymer donor materials based on poly(p-phenylene vinylidene) (PPV) units. The widespread use of aromatic imide repeating units, e.g., Naphthalimide (NDI) and Perylenediimide (PDI), has led to significant advances in the power conversion efficiency (PCE) of all-PSC devices. Nevertheless, the fill factor (FF) of all-polymer solar cell devices is relatively low (Figure 1) compared to their fullerene or small molecule acceptor counterparts, mainly due to complicated morphology control. Even the highly efficient devices developed in the past two years have difficulty in surpassing 70% FF.

The current molecular design strategy, namely, polymerizing the high-performance small molecular acceptor, led to a surge of polymer acceptors with excellent photovoltaic performance, which significantly improved device efficiency in this field. However, despite the fact that the state-of-the-art devices based on the polymerized small molecule acceptors (PSMAs) exhibit high open-circuit voltages (*V*_OC_s) and short circuit currents (*J*_SC_s), the FFs for a large fraction of them are relatively low due to the difference in the morphological and electrical properties between the polymer donor (high degree of polymerization) and the PSMA (low degree of polymerization). One of the solutions to this issue is optimizing the morphology and charge transport property, but for a given set of donor and acceptor materials, this can be difficult. From the experience of device engineering for organic photovoltaics in recent decades, another angle to tackle this problem, other than modifying the active layer directly, is interface engineering. So far, most high-performance all-PSCs utilize N,N′-Bis(N,N-dimethylpropan-1-amine oxide)perylene-3,4,9,10-tetracarboxylic diimide (PDINO), MoO_x_, or poly [(9,9-bis(3′-(N,N-dimethylamino)propyl)2,7-fluorene)-alt-5,5′-bis(2,2′-thiophene)-2,6-naphthalene1,4,5,8-tetracaboxylic-N,N′-di(2-ethylhexyl)imide] (PNDIT-F3N) as the electron transport material (ETM). Among them, PNDIT-F3N and its bromide version PNDIT-F3N-Br, are the workhorse ETMs in small molecule-based OSCs. However, the FFs of PNDIT-F3N-based all-PSCs are generally not high (hardly more than 70%, see Figure 1), which limits the further improvement of device efficiency [23,24,25,26,27,28,29,30,31,32,33,34,35,36]. For instance, Ref. [37] studied PM6:PYF-T devices with an efficiency of 14.10% and an FF of 67.73%. Ref. [38] researched PM6:PY-IT-based all-PSCs and reported an efficiency of 15.15% and an FF of 67.70% [39]. In 2020, Li’s group reported a new PDI-derived electron transport material, aliphatic amine-functionalized perylene-diimide (PDINN), which showed better contact with non-fullerenes active layers and better conductivity. The enhanced interfacial stability and higher conductivity, as well as the ability to reduce the work function of the metal cathode, make it more suitable for use as an electron transport material. In addition, PDINN is simple to synthesize and could be synthesized in large quantities by a one-step reaction. Therefore, PDINN is a low-cost alternative ETM for OSCs and has great promise for future large-scale production. Since its first report, PDINN has been most utilized in polymer:small molecule acceptor OSCs and proved effective in different cases, but it has not been adopted in all-PSCs with PCEs >16%.

In this work, we report all-PSCs with PM6 as the donor, PYF-T-*o* or PY-IT as the acceptor, and PNDIT-F3N or PDINN as the electron transport material. We systematically compare the performance of the all-PSC devices with PDINN and PNDIT-F3N, and we show that the device performance for PDINN-based devices is higher than those of PNDIT-F3N-based ones. Particularly, we show that the FF of the devices increases from 69.21% (PNDIT-F3N) to 72.05% (PDINN) for the PY-IT-based all-PSCs, with a corresponding efficiency increase from 15.47% to 16.41%, which is the highest power conversion efficiency (PCE) for PY-IT based binary all-PSCs. Through different characterizations, e.g., transient photocurrent and transient photovoltage, we find that the difference in their optical properties does not contribute much to the device performance variation. Instead, we attribute the main increase, i.e., the FF, of the PDINN-based devices, to the faster charge extraction and enhanced charge carrier lifetime, which are observed in both all-PSC systems we studied.

## 2. Experiments

### 2.1. Materials

Poly[(2,6-(4,8-bis(5-(2-ethylhexyl-3-fluoro)thiophen-2-yl)-benzo [1,2b:4,5-b′]dithio-phene))-alt-(5,5-(1′,3′-di-2-thienyl-5′,7′-bis(2-ethylhexyl)benzo [1′,2′c:4′,5′c′] dithio-phene-4,8-dione)] (PM6) was purchased from Dongguan Volt Ampere Photo-electric Technology Co., Ltd. (Dongguan, China). Poly[(2,2′-((2Z,2′Z)-((12,13-bis(2-octyldodecyl)-3,9-diundecyl-12,13-dihydro [1,2,5]thiadiazolo [3,4e]thieno [2″,3″:4′,5′]thieno [2′,3′:4,5] pyrrolo [3,2-g]thieno [2′,3′:4,5]thieno [3,2-b]-in-dole-2,10-diyl)bis(methanylylidene))bis(5-methyl-3-oxo-2,3-dihydro-1Hindene-2,1-diyl-idene)) dimalononitrile-co-2,5-thiophene (PY-IT) and N,N’-Bis{3-[3-(Dimethylamino)propylamino]propyl}perylene-3,4,9,10-tetracarboxylic diimide (PDINN) were purchased from Solarmer Materials Inc. (Beijing, China). Poly[(9,9-bis(3′-(N,N-dimethylamino)pro-pyl)2,7-fluorene)-alt-5,5′-bis(2,2′-thiophene)-2,6-naphthalene1,4,5,8-tetracabox-ylic-N,N′-di(2-ethylhexyl)imide] (PNDIT-F3N) and PYF-T-*o* were purchased from eFlexPV Limited. Poly(3,4-ethylenedioxythiophene) polystyrene sulfonate (PEDOT:PSS) (Clevios P VP 4083) were purchased from Heraeus Inc. (Hanau, Germany). All the other reagents and chemicals were purchased from Sigma Aldrich or Aladdin (Burlington, MA, USA and Shanghai, China) and used as received. Purity of solvents: chloroform (>99.8%), methanol (>99.5%), and acetic acid (>99.5%).

Solubility of PDINN and PNDIT-F3N:

PDINN: the solubility was 26.7 mg/mL in methanol without the assistance of any acid [40].

PNDIT-F3N: the solubility was >30 mg/mL in common organic solvents [41].

### 2.2. Device Fabrication

Organic solar cells were fabricated in a conventional device configuration of ITO(50 nm)/PEDOT:PSS(30 nm)/active layer(100~150 nm)/ETL(PNDIT-F3N or PDINN)(5~10 nm)/Ag(100 nm), as shown in the SEM image in Figure S3. The patterned indium tin oxide(ITO) glass was scrubbed with detergent and then sonicated with deionized water, acetone, and isopropanol sequentially and baked overnight in an oven. The glass substrate was treated whit UV-Ozone for 10 min before use. PEDOT:PSS solution was spin-casted onto them at 5200 rpm for 20 s, then dried at 150 °C for 10 min in air.

The different kinds of devices:

1. The PM6:PYF-T-*o* blend (1:1.2 weight ratio) was dissolved in chloroform (the concentration of donor was 6 mg mL^−1^ for all blends) with 1-chloronaphthalene (1% vol) as an additive and stirred overnight in a nitrogen-filled glove box. The blend solution was spin-casted at 2500 rpm for 30 s onto the PEDOT:PSS films, followed by a thermal annealing step at 95 °C for 5 min.
2. The PM6:PY-IT blend (1:1.2 weight ratio) was dissolved in chloroform (the concentration of donor was 6 mg mL^−1^ for all blends) with 1-chloronaphthalene (1% vol) as an additive and stirred overnight in a nitrogen-filled glove box. The blend solution was spin-casted at 2700 rpm for 30 s onto the PEDOT:PSS films, followed by a thermal annealing step at 95 °C for 5 min.

For both types of devices, either methanol with 0.5% vol acetic acid blend solution of PNDIT-F3N at a concentration of 0.5 mg mL^−1^ or a pure methanol solution of PDINN at a concentration of 1.0 mg mL^−1^ was spin-coated onto the active layer, respectively, at 2000 rpm for 30s and 3000 rpm for 30 s as the electron transport layer (ETL). Around 100 nm of Ag were evaporated under 1 × 10^−4^ Pa through a shadow mask. Then, encapsulation was carried out.

### 2.3. Characterization

The current density–voltage (*J*-*V*) curves of the PSCs were measured using a Keithley 2400 Source Meter under AM 1.5 G (100 mW cm^−2^) using an Enlitech solar simulator. The light intensity was calibrated using a standard Si diode with a KG5 filter to bring spectral mismatch to unity. An optical microscope (Olympus BX51) was used to define the device area (7.2 mm^2^) in a glove box filled with nitrogen (oxygen and water contents are smaller than 0.1 ppm). EQEs were measured using an Enlitech QE-S EQE system equipped with a standard single-crystal Si photovoltaic cell. Monochromatic light was generated from an Enlitech 300 W lamp source.

Transient photovoltage (TPV) and transient photocurrent (TPC) measurements: In TPV measurements, the devices were placed under background light bias enabled by a focused Quartz Tungsten-Halogen Lamp with an intensity of similar to working devices, i.e., the device voltage is close to the *V*_OC_ under solar illumination conditions. Photo-excitations were generated with 8 ns pulses from a laser system (Oriental Spectra, NLD520, Hyderabad, India). The wavelength for the excitation was tuned to 518 nm with a spectral width of 3 nm. A digital oscilloscope was used to acquire the TPV signal at the open-circuit condition. TPC signals were measured under short-circuit conditions under the same excitation wavelength without background light bias.

## 3. Results and Discussion

**Figure 2:**
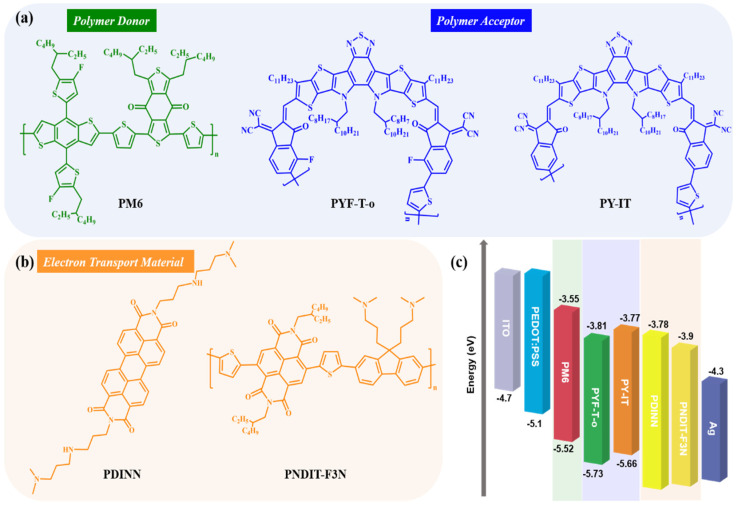
(**a**) Chemical structures of PM6, PYF-T-*o* and PY-IT. (**b**) Chemical structures of PDINN and PNDIT-F3N. (**c**) Energy diagram of materials (literature data: the energy levels of ITO, PEDOT: PSS, PM6, PYF-T-*o*, PNDIT-F3N, and Ag are taken from ref. [41], PY-IT from ref. [42], and PDINN from ref. [39]).

The chemical structural formulas of the donor PM6 and the acceptors PYF-T-*o* and PY-IT are shown in Figure 2a. The chemical structural formulas of the electron transport materials (PDINN and PNDIT-F3N) are shown in Figure 2b. Figure 2c shows the highest occupied molecular orbital (HOMO) and the lowest unoccupied molecular orbital (LUMO) energy levels of the active layer materials, as well as the energy level diagrams of the electron transport materials of PDINN and PNDIT-F3N.

**Figure 3:**
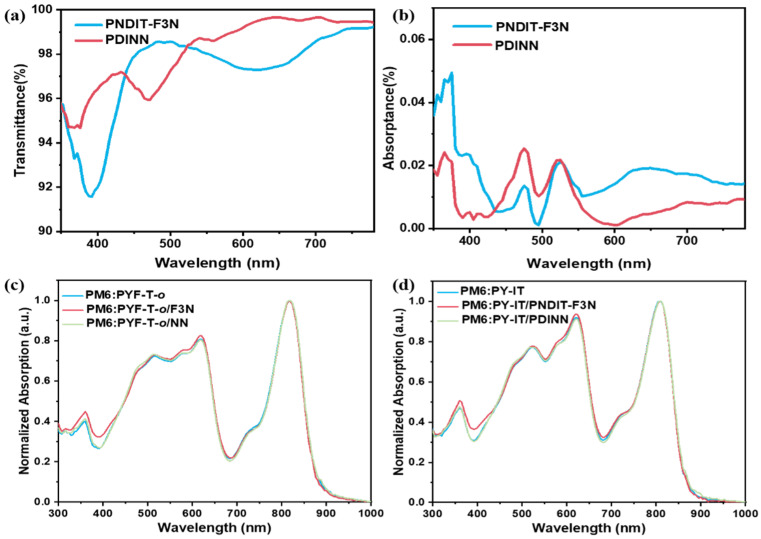
(**a**) UV-Vis transmittance spectra of PNDIT-F3N and PDINN. (**b**) UV-Vis absorption spectra of PNDIT-F3N and PDINN. (**c**,**d**) Absorption spectra of the active layers based on PYF-T-*o* and PY-IT, respectively.

To study the optical property of the two different interfacial layers, we measured the transmittance and absorption of PNDIT-F3N and PDINN. To mimic the thickness of PNDIT-F3N and PDINN used in devices, we used identical spin-coating parameters to prepare the PNDIT-F3N and PDINN films. From the absorption and transmittance curves (Figure 3a,b), we found that PNDIT-F3N showed absorption in the UV and visible regions. For instance, the absorption of the PNDIT-F3N film, despite being weak, peaked at ~390 nm. In addition, PNDIT-F3N also showed absorption in the range of ~570–650 nm. These absorptions of PNDIT-F3N can be reflected in the overall absorption spectra of active layer/PNDIT-F3N, which is shown in Figure 3c,d.

**Figure 4:**
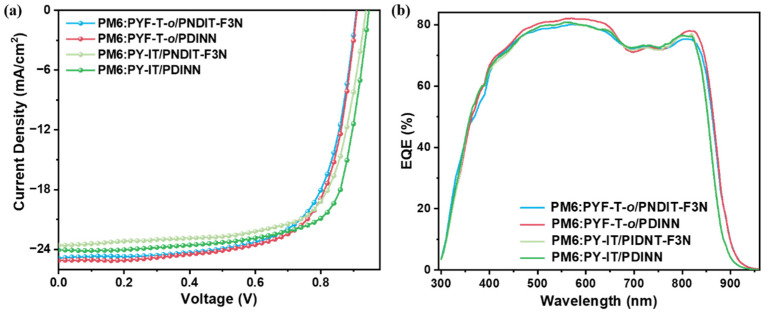
(**a**) *J-V* curves and (**b**) the corresponding EQE spectra of all-PSCs based on PM6 and PYF-T-*o* or PY-IT.

For the PYF-T-*o*-based devices, the difference between *V*_OC_ and *J*_SC_ of PNDIT-F3N and PDINN devices was not significant. The main difference derived from the change in FF, where PDINN improved the filling factor of the device from 68.30% to 69.90% for PNDIT-F3N. For the PY-IT-based devices, PNDIT-F3N and PDINN had little effect on the *J*_SC_ of the device, and the main difference continued to come from the significant increase in the fill factor, for which PDINN increased from 69.21% of PNDIT-F3N to 72.05% of the device. As a result, these improvements significantly increased the efficiency of the PDINN-based devices by 16.41%. The corresponding specific device performance parameters are listed in Table 1 and Tables S2 and S3. We then conducted EQE, as shown in Figure 4b. From the EQE curves, we found that the current obtained from the EQE integration is consistent with the *J*_SC_ obtained from the *J-V* test (Figure 4a).

**Figure 5:**
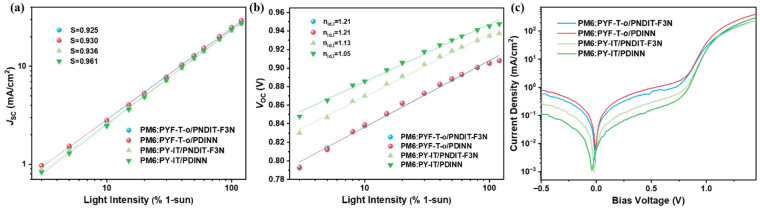
(**a**,**b**) are light intensity dependence of *J*_SC_ and *V*_OC_ of the OSCs based on PM6:PYF-T*-o* and PM6:PY-IT, respectively. (**c**) Dark *J-V* characteristics of the OSCs.

To investigate recombination, we first measured the *J-V* characteristics of the four devices under different light intensities (*I*). Figure 5a plots the relationship between *J*_SC_ and light intensity. By linearly fitting the *J*_SC_ versus light intensity data, we obtained the slope (*S*) for the four devices. The *S* values for the PM6:PYF-T-*o*/PNDIT-F3N, PM6:PYF-T-*o*/PDINN, PM6:PY-IT/PNDIT-F3N, and PM6:PY-IT/PDINN devices are 0.925, 0.930, 0.936, and 0.961, respectively. It is known that the closer *S* is to unity, the weaker the bimolecular recombination. Therefore, the smallest *S* for PM6:PY-IT/PDINN suggests the weakest bimolecular recombination in it, and among the four different devices, the trend of *S* is consistent with the trend of the FF of the devices.

To access trap-assisted recombination, we plot the *V*_OC_ versus light intensity result in Figure 5b. By fitting the *V*_OC_ versus ln(*I*) curves, we obtained the ideality factor, *n*_id,l_, from the equation $n_{\mathrm{id},l}=\frac{q}{kT}\frac{\partial V_{oc}}{\partial \ln\left(I\right)}$. The *n*_id_._l_s of the PM6:PYF-T-*o*/PNDIT-F3N, PM6:PYF-T-*o*/PDINN, PM6:PY-IT/PNDIT-F3N, and PM6:PY-IT/PDINN devices are 1.21, 1.21, 1.13, and 1.05, respectively. From diode theory, higher *n*_id,__l_ means that trap-assisted recombination is stronger. Therefore, PM6:PY-IT/PDINN has the weakest trap-assisted recombination (*n*_id__,l_ = 1.05) among the four devices, which agrees with its highest FF.

Another method to obtain the ideality factor is to fit the exponential region of the dark *J-V* curve. We measured the dark *J-V* curves for the devices, and the results are shown in Figure 5c. From the fitting, the *n*_id,d_ of the PM6:PYF-T-*o*/PNDIT-F3N, PM6:PYF-T-*o*/PDINN, PM6:PY-IT/PNDIT-F3N, and PM6:PY-IT/PDINN devices are 1.823, 1.719, 1.642 and 1.582, respectively. The difference between the magnitude of *n*_id,l_ and *n*_id,d_ is detailed elsewhere [43], but the trend of the *n*_id,d_ is overall consistent with that of *n*_id,l_.

**Figure 6:**
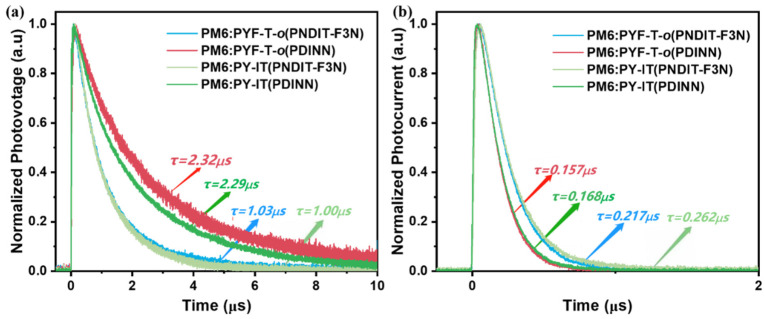
TPV (**a**) and TPC (**b**) decays of the PM6:PYF-T-*o* and PM6:PY-IT-based devices.

To further study the charge recombination and charge extraction, we performed TPV and TPC measurements. The details of the experimental setup for these measurements can be found in the experimental section. As shown in Figure 6a, we fitted the decay using a monoexponential function, which revealed that the decay constants have the following relationship, τ_PDINN_ > τ_PNDIT-F3N_, indicating that the charge carrier lifetime is longer in devices based on PDINN than in PNDIT-F3N-based devices. This indicates that the recombination in the PDINN-based devices is weaker than that in the PNDIT-F3N-based devices. One hypothesis is that the PDINN has better/higher surface coverage than PNDIT-F3N so the contact between the active layer and cathode is reduced in PDINN-based devices, which reduces surface recombination and improves FF. In addition, from the TPC measurements (Figure 6b) for both PYF-T-*o* and PY-IT-based devices, the charge extraction in the PDINN-based devices is significantly faster than in the PNDIT-F3N-based devices. This indicates that the charge collection efficiency of PDINN is higher than that of PNDIT-F3N, which could be one of the determining factors of the high FF of the PNDIT-F3N devices.

**Figure 7:**
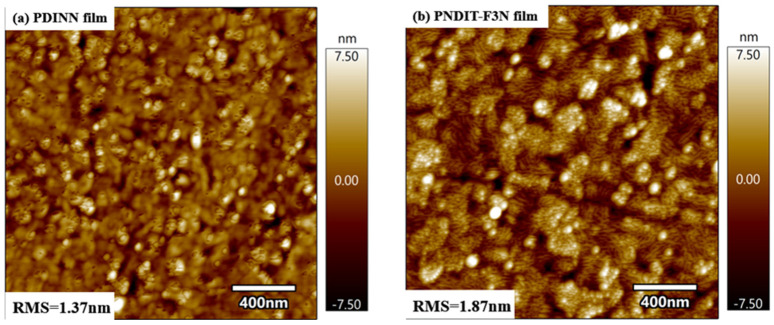
AFM images of PDINN and PNDIT−F3N.

By analyzing the results of AFM (Figure 7), the RMS of the PDINN film is 1.37 nm, and that of PNDIT-F3N is 1.87 nm. Leaving energetics alone, just from a morphological point of view, the smoother surface of PDINN is beneficial for obtaining better coverage on the active layer, which could then reduce the direct contact between the active layer material and the metal electrode. This could reduce surface recombination, protect the active layer from hot metal penetration or reaction during evaporation, increase device shunt resistance, and enhance the FF of the device.

## 4. Conclusions

In conclusion, we addressed the relatively low FF in PY-IT and PYF-T-*o*-based all-polymer solar cells through active layer-cathode interface engineering. Specifically, we used PDINN as an electron transport layer material in PM6:PYF-T-*o* and PM6:PY-IT-based devices, compared it with the widely employed, high-performance ETM, PNDIT-F3N, compared its photovoltaic performance, and investigated the charge extraction and recombination. It was found that the PDINN-based devices demonstrated faster charge extraction and longer charge carrier lifetime compared to PNDIT-F3N devices. Consequently, we demonstrated that PDINN could effectively promote the FF of the all-PSC devices studied in this work and thus improve the PCE of the devices. Particularly in the PM6:PY-IT-based device, the FF increased from 67.99% (PNDIT-F3N) to 72.05% (PDINN), and the PCEs increased from 15.47% to 16.41%, which is the highest efficiency reported to date for PY-IT-based binary all-polymer solar cells.

## Figures and Tables

**Table 1:** Photovoltaic parameters of the solar cell devices based on PM6:PYF-T-*o* and PM6:PY-IT under AM 1.5 G illumination at 100 mW cm^−2^.

Devices	*V*_OC_ (V)	*J*_SC_ (mA/cm^2^)	FF (%)	PCEs (%)	*S*	*n*_id,l_	*n*_id,d_
PM6:PYF-T-*o*/ PNDIT-F3N	0.913 *^a^* (0.910 ± 0.002)	24.91 (24.9 ± 0.53)	68.30 (66.7 ± 0.96)	15.47 (15.1 ± 0.27)	0.925	1.21	1.82
PM6:PYF-T-*o*/PDINN	0.908 (0.907 ± 0.003)	24.83 (24.8 ± 0.31)	69.90 (69.1 ± 0.78)	15.78 (15.5 ± 0.17)	0.930	1.21	1.71
PM6:PY-IT/PNDIT-F3N	0.938 (0.938 ± 0.005)	23.85 (23.6 ± 0.47)	69.21 (67.8 ± 0.83)	15.47 (15.1 ± 0.29)	0.936	1.13	1.62
PM6:PY-IT/PDINN	0.950 (0.951 ± 0.002)	23.95 (24.0 ± 0.17)	72.05 (70.5 ± 0.89)	16.41 (16.1 ± 0.17)	0.961	1.05	1.58

*^a^* Parameters for devices with the highest PCEs. Values in brackets are average values and standard deviations based on 10 independent devices.

## Supplementary Materials

The following supporting information can be downloaded at: https://www.mdpi.com/article/10.3390/polym14183835/s1.
