# Supplementary material for: Efficient All-Polymer Solar Cells Enabled by Interface Engineering
Source: Polymers (Basel). 2022 Sep 14;14(18):3835. doi: 10.3390/polym14183835 (PMC9505650; doi:10.3390/polym14183835)
Supplement: Supplementary file 1 [file polymers-14-03835-s001.zip › polymers-1902872-supplementary.pdf]

SI:

Efficient All-Polymer Solar Cells Enabled by Interface Engineering

Guoping Zhang[1†], Lihong Wang[1†], Chaoyue Zhao[1†], Yajie Wang[1], Ruiyu Hu[1], Jiaxu Che[1], Siying He[1], Wei Chen[2,*], Leifeng Cao[2], Zhenghui Luo[3,*], Mingxia Qiu[1,*], Shunpu Li[1,*] and Guangye Zhang[1,*]

[1] College of New Materials and New Energies, Shenzhen Technology University, Shenzhen 518118, China

[2] College of Engineering Physics, Shenzhen Technology University, Shenzhen 518118, China

[3] College of Materials Science and Engineering, Shenzhen University, Shenzhen 518060, China

* Correspondence: chenwei@sztu.edu.cn, zhhuiluo@szu.edu.cn, qiumingxia@sztu.edu.cn, lishunpu@sztu.edu.cn, zhangguangye@sztu.edu.cn.

† These authors contributed equally to this work.

Device characterization.

SCLC Measurements: The electron- and hole-mobilities were measured using space charge limited current (SCLC) method. The device architecture of the electron-only devices were ITO/PNDIT-F3N/active layer/PNDIT-F3N/Ag and ITO/PDINN/active layer/PDINN/Ag. The device architecture of the hole-only devices was ITO/PEDOT:PSS/active layer/Au. The charge carrier mobilities were determined by fitting the dark current into the model of a single carrier SCLC according to the equation: $J = 9\varepsilon_0\varepsilon_r\mu V^2/8d^3$, where J is the current density, d is the film thickness of the active layer, μ is the charge carrier mobility, ε_r is the relative dielectric constant of the transport medium, and ε_0 is the permittivity of free space. $V = V_{app} - V_{bi}$, where V_{app} is the applied voltage, V_{bi} is the offset voltage. The carrier mobilities were calculated from the slope of the J~ V^2 curves.

Table S1 EQE, μ_h and μ_e of PM6:PYF-T-*o*/PNDIT-F3N,PM6:PYF-T-*o*/PDINN,PM6:PY-IT/PNDIT-F3N and PM6:PY-IT/PDINN.

Samples	J_{EQE} (mA/cm^2)	μ_h [cm^2 V^{-1} s^{-1}]	μ_e [cm^2 V^{-1} s^{-1}]
PM6:PYF-T-*o*/PNDIT-F3N (BHJ)	23.90	1.83E-04	1.34E-04
PM6:PYF-T-*o*/PDINN (BHJ)	24.30	1.83E-04	1.76E-04
PM6 :PY-IT/PNDIT-F3N (BHJ)	23.45	2.65E-04	1.17E-04
PM6 :PY-IT/PDINN (BHJ)	23.51	2.65E-04	2.91E-04

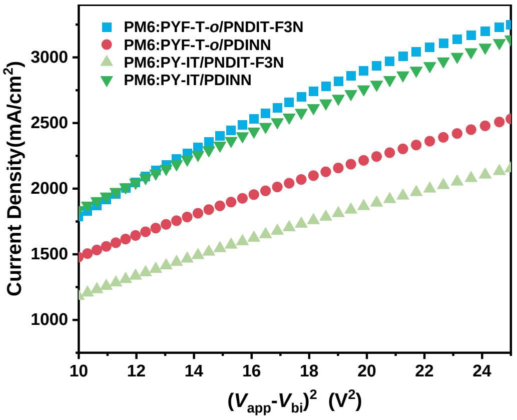

Figure S1 Electron mobility curves of PM6:PYF-T-*o*/PNDIT-F3N,PM6:PYF-T-*o*/PDINN,PM6:PY-IT/PNDIT-F3N and PM6:PY-IT/PDINN.

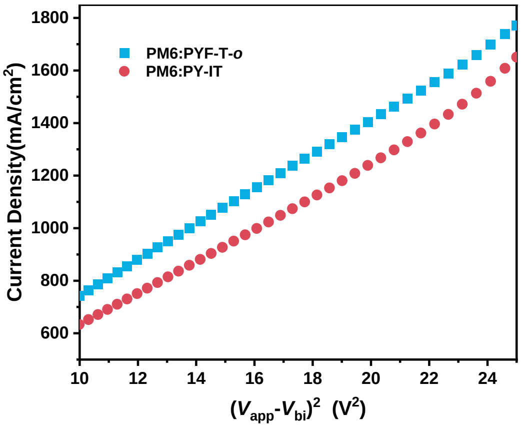

Figure S2 Hole mobility curves of PM6:PYF-T-*o* and PM6:PY-IT.

Table S2 Device optimization: The spin coating parameters of active layer are compared.

Devices	V_{OC} [V]	J_{SC} [mA/cm^2]	FF [%]	PCE [%]
PM6:PYF-T-o(2000rpm) / PNDIT-F3N	0.910	24.4	66.8	14.8
PM6:PYF-T-o (3000rpm) /PNDIT-F3N	0.912	23.8	66.8	14.4
PM6:PYF-T-o(2000rpm) /PDINN	0.911	24.6	65.6	14.8
PM6:PYF-T-o (3000rpm) /PDINN	0.912	23.8	66.4	14.5
PM6:PYIT(2500rpm) /PNDIT-F3N	0.911	24.1	66.4	14.6
PM6:PYIT(3000rpm) /PNDIT-F3N	0.911	24.1	66.4	14.6
PM6:PYIT(2500rpm) /PDINN	0.931	23.9	69.2	15.4
PM6:PYIT(3000rpm) /PDINN	0.931	23.9	70.1	15.6

Table S3 Device optimization results. Different ETL processing conditions are compared.

Devices	V_{OC} [V]	J_{SC} [mA/cm²]	FF [%]	PCE [%]
PM6:PYF-T-*o*/ PDINN（0.5mg/ml、1500rpm)	0.898	24.766	65.321	14.443
PM6:PYF-T-*o*/ PDINN（0.5mg/ml、2000rpm)	0.902	25.250	65.057	14.725
PM6:PYF-T-*o*/ PDINN（0.5mg/ml、2500rpm)	0.908	23.563	67.586	14.365
PM6:PYF-T-*o*/ PDINN（1.0mg/ml、2500rpm)	0.912	25.640	65.712	15.266
PM6:PYF-T-*o*/ PDINN（1.0mg/ml、3000rpm)	0.907	25.856	66.660	15.527
PM6:PYF-T-*o*/ PDINN（1.0mg/ml、3500rpm)	0.907	25.091	66.863	15.119
PM6:PYF-T-*o*/ PDINN（2mg/ml、3000rpm)	0.911	24.036	66.253	14.518
PM6:PYF-T-*o*/ PDINN（2mg/ml、4000rpm)	0.912	24.735	66.625	15.027
PM6:PYF-T-*o*/ PDINN（2mg/ml、5000rpm)	0.913	23.408	68.307	14.598
PM6:PYF-T-*o*/ PDINN（3mg/ml、4000rpm)	0.910	24.026	67.046	14.669
PM6:PYF-T-*o*/ PDINN（3mg/ml、5000rpm)	0.912	24.780	66.933	15.123
PM6:PYF-T-*o*/ PDINN（3mg/ml、6000rpm)	0.911	24.103	66.170	14.529

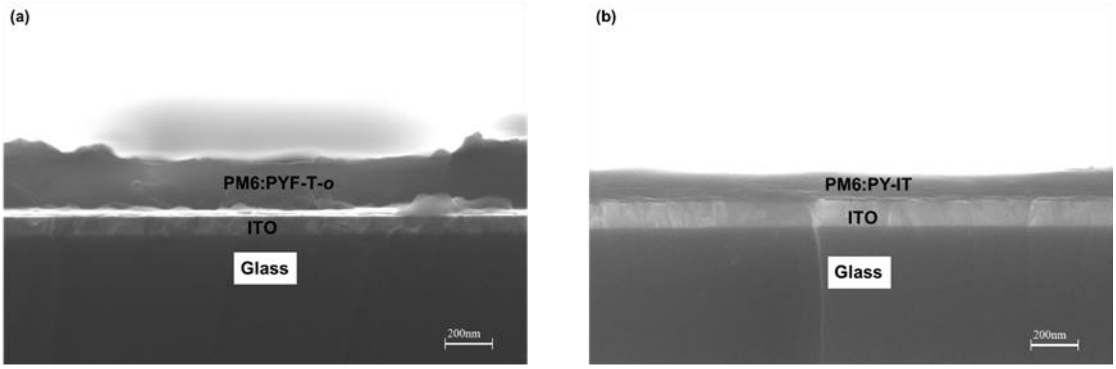

Figure S3. cross-sectional SEM image of each layar.
